# Management of Pediatric Elbow Fractures and Dislocations

**DOI:** 10.3390/children11080906

**Published:** 2024-07-27

**Authors:** Marko Bašković, Domagoj Pešorda, Luca Zaninović, Damir Hasandić, Katarina Lohman Vuga, Zenon Pogorelić

**Affiliations:** 1 Department of Pediatric Surgery, Children’s Hospital Zagreb, Ulica Vjekoslava Klaića 16, 10000 Zagreb, Croatia; 2 School of Medicine, University of Zagreb, Šalata 3, 10000 Zagreb, Croatia; 3 Scientific Centre of Excellence for Reproductive and Regenerative Medicine, School of Medicine, University of Zagreb, Šalata 3, 10000 Zagreb, Croatia; 4 Department of Pediatric Surgery, Clinical Hospital Center Rijeka, Vjekoslava Dukića 7, 51000 Rijeka, Croatia; 5 School of Medicine, University of Rijeka, Braće Branchetta 20, 51000 Rijeka, Croatia; 6 Special Hospital for Medical Rehabilitation Varaždinske Toplice, Trg Svetog Martina 1, 42223 Varaždinske Toplice, Croatia; 7 Department of Pediatric Surgery, University Hospital of Split, Spinčićeva ulica 1, 21000 Split, Croatia; 8 School of Medicine, University of Split, Šoltanska ulica 2a, 21000 Split, Croatia

**Keywords:** pediatric elbow fractures, pediatric elbow dislocations, supracondylar humeral fracture, lateral condyle fracture, medial condyle fracture, lateral epicondyle fracture, medial epicondyle fracture, T-condylar fracture, proximal radius fracture, olecranon fracture

## Abstract

Pediatric elbow fractures and dislocations have always been a challenge from a diagnostic and therapeutic point of view, primarily due to the complex nature of the pediatric elbow, especially its developmental anatomy. They must be diagnosed and treated on time to prevent numerous complications with long-term consequences. With the development of radiology and pediatric surgery and orthopedics, as well as the development of modern osteosynthesis materials, concerning current scientific and professional knowledge, the outcomes are getting better, with fewer acute and chronic complications. This comprehensive review aims to provide clinicians current knowledge about pediatric elbow fractures and dislocations so that in daily practice they have as few doubts as possible with the best possible treatment outcomes.

## 1. Introduction

Elbow fractures are among the most common injuries, right after fractures of the distal part of the forearm [1,2]. Elbow fractures account for 5–10% of all fractures in the pediatric population, and in some series they account for up to 85% of surgically treated pediatric injuries [3]. Although relatively common, these injuries can be difficult to diagnose and consequently treat [1,4]. Although there is variation in timing, there is a predictable pattern of ossification in the pediatric elbow. The patterns of ossification of the elbow are well known and follow the CRITOE mnemonic, in which the first center to ossify is the capitellum at an average of 3 months, followed by the radial head at 5 years, the medial epicondyle at 7 years, the trochlea at 9 years, the olecranon at 11 years and the lateral epicondyle at the age of 13. It is generally considered that fusion of elbow growth centers completed by age 13 in girls and age 15 in boys [5,6]. Regarding range of motion with growth, differences in pediatric elbow range of motion between girls and boys are not clinically significant [7].

### 1.1. Physical Examination

Pediatric elbow injuries often present a diagnostic dilemma because of their unique anatomy with a narrow therapeutic window and relatively high complication rates associated with certain fracture types [8]. Acute fractures are usually associated with high-energy trauma or a fall from a height onto an outstretched arm [9,10]. Although most injuries are isolated elbow fractures, in these situations it is important to consider possible associated neurovascular injuries, which can complicate the overall treatment. Knowledge of the normal growth pattern is extremely important for proper evaluation and treatment of these injuries. In general, the younger the child at the time of the injury, the more difficult the assessment may be [11]. With chronic injuries, it is important to ask questions about participation in sports. This includes an accurate description regarding the type of activity and duration [12].

During the clinical examination, it should be determined whether there are ecchymosis, scratches, tissue loss or swelling on the skin that indicate an acute injury. Signs of an open fracture such as small puncture sites, occasional oozing or open bleeding, and exposed bone must also be evaluated. In patients with chronic symptoms, long-term changes such as hypertrophy or atrophy of the surrounding musculature and possible joint contractures should be evaluated. Areas of tenderness to palpation may indicate a fracture and require further investigation. Palpation of the soft tissues of the upper arm and forearm is useful in case of severe trauma, considering the possible development of compartment syndrome [1,13]. Normal range of motion in the flexion-extension plane is generally described as 140° of flexion to extension of 0 to −10° of hyperextension, with the assessment made with the patient holding the forearm supinated. Normal forearm pronation and supination are typically 75° and 85°, respectively [14,15]. In the event of a high-energy injury, but even after isolated trauma to the elbow, a thorough neurovascular examination must be performed [1]. A thorough motor and sensory examination should focus on the median, ulnar, and radial nerves. Vascular assessment is critical to distinguish between the normal exam, the limb that has perfusion but is pulseless, and the dysvascular hand [16,17].

### 1.2. Radiographic Evaluation

In addition to the clinical challenge of diagnosis, the interpretation of radiographs of an injured child’s elbow is even more difficult. Considering the development and ossification centers that change from year to year, we often witness errors in diagnosing injuries [18]. Initial radiographs should include an anteroposterior (AP) and lateral view of the elbow. Separate imaging of the humerus and forearm is often necessary to reveal the full extent of potential injuries. To limit exposure to ionizing radiation, images of the contralateral elbow are not part of the routine evaluation unless comparative views are deemed necessary to evaluate whether a possible abnormal finding is a fracture, ossification center, or growth variation [19]. AP images are captured with the forearm in full supination and the elbow joint in as much extension as tolerated by each child. Lateral views should be taken with the elbow joint flexed to 90° and the forearm in maximal supination [20]. From the lateral view, it is necessary to pay attention to the anterior and posterior fat pads. Although an anterior fat pad is a normal finding, an effusion present in the joint displaces the fat more anteriorly, which ultimately changes the radiographic appearance of this fat pad (sail sign) which may indicate an occult fracture. A positive posterior fat pad sign should always be considered abnormal, i.e., indicative of a non-displaced, occult fracture [21,22].

Regarding the lines from the lateral projection, it is important to mention the anterior humeral line (AHL), which usually intersects the middle third of the capitellum. Disturbance, even a minimal forward displacement of the line far from the expected location for age, indicates a posterior displacement of extension supracondylar humerus fracture in children [23,24]. Radiocapitellar line is another important landmark. In most children older than 2 years, a horizontal line drawn along the axis of the radius, and especially the radial neck, normally cuts through the middle of the capitellum, regardless of the position of the elbow or forearm. This radiographic line is very useful in identifying Monteggia fractures in children [18,19,24]. In relation to the AP projection, it is important to separate the humeroulnar line (carrying angle) and Baumann’s angle (humerocapitellar angle). The carrying angle of the elbow has an important clinical meaning and represents the normal degree of angle that exists in this joint. This angle varies depending on age and gender, but in most children and adolescents it ranges from 5° to 20° valgus. A humeroulnar angle of less than 5° is considered abnormal, and any patient with an angle greater than zero is said to have *cubitus varus*. A humeroulnar angle greater than 20° is also considered abnormal and is called *cubitus valgus* [19,24,25]. Baumann’s angle is formed by the intersection of a vertical line that bisects the distal humerus, with a horizontal line along the center of the ossification of the lateral condyle. The normal value can vary between 64° and 81°. Fractures that cause varus or internal rotation will increase this angle [18,24,26].

## 2. Supracondylar Humerus Fractures

Supracondylar humerus fracture is the most common fracture in the elbow area, and the second most common of all pediatric fractures, right after the fracture of the distal radius. They most often appear between the ages of five and seven, without gender differences [9,27,28]. Considering the relatively thin cortex in the supracondylar region, especially in the age group around the age of six when remodeling occurs in the said region, it is at high risk for fracture [29,30]. Considering the relatively small longitudinal growth of the distal humerus region, this region has little remodeling potential, in contrast to the proximal part of the humerus [31,32].

### 2.1. Classification

Supracondylar fractures of the humerus can be flexion or extension depending on the direction of displacement of the distal fragment. The flexion type is extremely rare (usually caused by a direct blow to the elbow), while the extension type accounts for 97–99% of fractures (usually caused by a fall on an outstretched arm). Extension fracture types are usually classified using the modified Gartland classification system [33,34,35] (Table 1).

### 2.2. Clinical Presentation and Radiographic Evaluation

Children with a supracondylar humerus fracture usually report pain and swelling around the elbow that may extend into the forearm with limited mobility [29]. Along with a basic visual examination of the region, the skin folds and the presence of ecchymosis in the antecubital fossa indicate a more extensive soft tissue injury due to a bony fragment that may pierce the brachialis muscle. The pucker sign (also called skin tenting, dimpling, or anterior spiking) indicates brachialis penetration by the proximal portion of the fracture and is a possible sign of entrapment of the median nerve and brachial artery. If bleeding from the wound is observed, it is usually an open fracture. By comparing the color and appearance of the skin of the limb, it is determined whether there is adequate perfusion distal to the site of injury [9,36]. By vascular assessment of the limb, we can usually determine whether it is of normal color, pink (limb with spasm or injury of the brachial artery but with sufficient collateral circulation) or white and without a pulse [37]. In addition to the vascular assessment, the sensory and motor function of each nerve (radial, median and ulnar) must also be assessed [9,29,38].

Radiologically, the elbow is evaluated in the AP and lateral projection of the elbow, with the fact that the lateral view is especially useful for evaluating a supracondylar fracture because the elbow may appear normal in the AP projection. Attention should also be paid to the presence of fat pads, which may indicate an occult fracture. Of the radiographic parameters, special attention should be paid to the anterior humeral line (Roger’s line) in the lateral projection if it does not cross the capitellum, and to Baumann’s angle in the AP projection. The AHL is the most reliable radiographic measure for quantifying anterior-posterior displacement in supracondylar humeral fractures and is important for achieving anatomical alignment. It was found that after the reduction of supracondylar fractures whose AHL passed in front of or through the anterior third of the capitellum, children had worse early elbow flexion and the total range of motion of the elbow. The normal Baumann angle ranges from 9° to 26°, and a Baumann angle less than 9° indicates varus angulation [9,24,39,40,41].

### 2.3. Management

#### 2.3.1. Non-Surgical

Gartland type I fractures are an indication for non-surgical treatment of a supracondylar humerus fracture. Treatment consists of immobilization with the elbow flexed 60°–90° for three weeks, followed by exercises with or without formal physical therapy [42,43]. Regarding the Gartland type II fracture, there is still no clear opinion. While closed reduction and casting are advocated on the one hand closed reduction and percutaneous fixation are advocated on the other hand [44,45,46,47,48]. Proponents of percutaneous fixation rely on research that indicates that in a large number of children, residual deformity is left behind if only closed reduction and casting are performed [49,50].

#### 2.3.2. Closed Reduction and Percutaneous Pinning

Indications for closed reduction and percutaneous pinning (CRPP) include type III and type II fractures if previous opinions are advocated (displaced fracture with AHL falling anterior to the capitellum or a reduced Baumann angle). Also, neurovascular compromise itself is an indication for CRPP [48,50,51,52]. Unless it is an open fracture, neurovascular failure, floating elbow or the evolving compartment syndrome, there is no evidence that urgent surgical treatment reduces the rate of perioperative complications in the form of pin track infection, iatrogenic nerve and vascular injury, compartment syndrome or the final functional status of the elbow. If the surgical treatment is delayed for more than 24 h, the use of skin or trans-skeletal traction is avoided by most surgeons, preferring fracture splinting [53,54,55,56]. Presently, the evidence supporting patient prone versus supine positioning is scarce. The choice of position is based on the surgeon’s experience. Prone position has been shown to facilitate reduction, use the C-arm more easily, and reduce the rate of iatrogenic nerve injury, but makes it difficult airway management and possible conversion to an open reduction when it is necessary to repositioned the patient. Most surgeons prefer the supine position because it is quicker, it allows for standard anesthesia management and it gives the possibility to perform an anterior, anteromedial, or anterolateral approach, if conversion to open reduction is required [56,57,58,59]. As far as the technique of surgical treatment is concerned, after sterile preparation, a “milking maneuver” is usually performed in order to disengage the brachialis and biceps brachii muscle fibers from the distal spike of the proximal fragment, if any. By positioning the fragments in the sagittal plane, it is necessary to access the coronary alignment. After fitting, the elbow should be held in hyperflexion to maintain alignment and assess reduction. If the anatomical reduction is difficult, it is possible that neurovascular structures have been pinched at the fracture site, in which case open reduction is necessary. After anatomical reduction, the fracture should be stabilized percutaneously by placing pins. Generally, two pins are set for type II fractures and three pins for type III fractures. In order to avoid iatrogenic injury to the ulnar nerve, lateral entry pins have been shown to have equal results compared to crossed pins, although some studies still advocate greater stability of crossed pins [60,61,62]. After the fracture is stable with a regular pulse, the pins are bent and cut. The elbow is then cast in a position of 45°–80° of elbow flexion [36,63,64,65]. Postoperative radiographs within three weeks do not affect the outcome, and three weeks after the procedure, the cast is removed and radiographic imaging is performed to see the callus formation. If the callus is sufficient, the pins are removed and the child is referred to usual activities with or without formal physical therapy to restore elbow range of motion [65,66,67].

#### 2.3.3. Open Reduction and Pinning

Indications for open approach are open, irreducible, both neurologically compromised (median nerve) and vascularly compromised, dysvascular limbs following closed reduction, pulseless limbs following reduction when a pulse was present preoperatively and limbs that remain pink and pulseless following CRPP [68,69,70]. From a surgical point of view, the anterior approach to the antecubital fossa is the most appropriate. After the skin incision, a dissection is made through the dermis and aponeurosis while paying attention to the neurovascular structures, identifying the median nerve and the brachial artery. If soft tissue is found inside the fracture, such as the brachialis muscle, it must be removed. If an injury to the brachial artery is detected, it is necessary to perform a direct repair or to transplant a venous bypass. Pins placement is the same as during CRPP [71,72].

### 2.4. Complications

Complications include possible loss of pulse or perfusion after CRPP, which suggests entrapment of the brachial artery. Pins should be removed immediately, and the brachial artery should be explored when converting to an open approach [73]. With an associated, ipsilateral forearm injury, the risk of compartment syndrome increases. Typical signs of compartment syndrome (pain, pallor, loss of pulse, paresthesia, paralysis) are not reliable in children, and the increasing need for analgesics is the most reliable indicator. If the evolving compartment syndrome is suspected, flexion should be reduced immediately and fasciotomies performed urgently if necessary [74,75]. Iatrogenic injuries to the ulnar nerve usually occur during medial pinning. If the above injury is found, the pin must be removed. Most injuries are neuropraxias and resolve spontaneously within 3 months [76]. As for the pins themselves, migration or infection of the pin site can usually occur, in which case, in case of infection, it is necessary to remove them and give the child an oral antibiotic. Migration is best prevented by leaving the pins 1 cm above the skin and bending the wire at 90° [75,77]. Technical errors when placing pins can lead to loss of reduction (less than two pins engaging both fragments, less than two bicortical pins, insufficient pin separation at the fracture site (<2 mm)) [78]. If a significant varus deformity remains, an osteotomy should be performed [79].

## 3. Distal Humerus Transphyseal Fracture

Transphyseal fractures of the distal humerus are rare and can be seen in children up to the age of three, usually caused by a fall. This type of fracture is most often detected by radiography when the axis of the forearm is no longer in line with the axis of the humerus. There is no official classification of these fractures, but most of them are Salter-Harris type 1 fractures, but sometimes they can also be Salter-Harris type 2 fractures [80,81,82].

### 3.1. Clinical Presentation and Radiographic Evaluation

This type of fracture is very often not recognized in time, and parents usually bring their children with a delay because they notice that the child is not using hand [81]. Physical examination can reveal swelling and redness, local sensitivity, pain or discomfort when trying to manipulate the child’s hand [83]. While radiography is sufficient for significant displacements, fractures with subtle displacement are difficult to recognize radiographically, and in these cases additional diagnostics in the form of ultrasound or magnetic resonance imaging are required. In the case of radiographically visible displacements, it is observed that the axis of the forearm is not in line with the axis of the humerus. In somewhat older children, a small bone fragment (Thurston-Holland fragment) can be observed, which facilitates the diagnosis [84,85,86].

### 3.2. Management

#### 3.2.1. Non-Surgical

Most of the cases described so far were treated non-surgically. Studies advocate closed reduction with 90° elbow flexion and forearm pronation if the fracture is stable. Given that these fractures are usually recognized late, when there is already abundant callus, non-surgical treatment is usually sufficient if there is no major deformity. An extremely small number of patients treated non-surgically developed *cubitus varus*, of which a smaller number required corrective osteotomy [87,88].

#### 3.2.2. Surgical

For dislocated unstable fractures, surgical treatment is indicated. Although several series with open reduction have been described so far, most authors favor closed reduction with lateral percutaneous pinning [89,90,91]. During fixation, care must be taken to ensure that there is no coronal plane malalignment or malrotation, as well as that the AHL intersects the capitellum [92]. Some surgeons prefer to do an arthrogram to better visualize the fracture. After satisfactory reduction in all planes and maintained hyperflexion, two K-wires are usually placed percutaneously laterally to avoid injury to the ulnar nerve. Three weeks after surgical treatment and the arm being in a cast, the pins are removed and further movements in the elbow in all directions are recommended in order to achieve a proper range of motion [1,90,92].

### 3.3. Complications

Given that missed, incorrect and late diagnoses are common in this type of fracture, there are also numerous complications. Special mention should be made of *cubitus varus* and *cubitus valgus*, which can be corrected by osteotomy. Also, although rare, avascular necrosis is also possible, for which there is still no adequate management opinion. In order to minimize complications, especially with less dislocated fractures of this type, it is necessary to appeal to surgeons not to hesitate to use additional diagnostics in the form of ultrasound and magnetic resonance imaging [90,91,93].

## 4. Lateral Condyle Fractures

After supracondylar fractures, lateral condyle fractures are the second most common. It is usually caused by falls on an outstretched arm with the forearm in a supinated position, a fall on the palm with a flexed elbow, or direct injuries to the elbow [94,95].

### 4.1. Classification

When looking at fractures of the lateral condyle there are several classifications. The first is Milch’s classification, which divides fractures into two types, type I and type II, depending on whether the fracture crosses the trochlear groove (Table 2). Also, type II fractures are associated with ulnar dislocation with rotation [95].

The following is Jakob’s classification, which divides fractures of the lateral condyle into incomplete, complete or complete with a rotated fractured fragment (Table 3) [96].

Finnbogason et al., fractures with displacement <2 mm were grouped into three groups (Table 4) [97].

Finally, Weiss et al. described a classification scheme based on displacement and articular surface congruency (Table 5) [98]. Currently, this classification system is the most accepted by surgeons worldwide.

### 4.2. Clinical Presentation and Radiographic Evaluation

Regarding the clinical examination of a child with a fracture of the lateral condyle, sensitivity to palpation at the fracture site is usually present, and ecchymosis is often present, the cause of which is a tear in the brachioradialis aponeurosis [95,99]. The initial method of radiological diagnosis is plain radiography in AP and lateral projection, with a note that an additional internal oblique projection can provide the best visualization. It is important to note and keep in mind that real displacement is usually greater than can be measured radiographically [100,101]. In severe cases, especially in younger children, an arthrogram can be used as an intraoperative diagnostic aid [102]. Compared to plain radiography, computed tomography can more accurately determine the magnitude of fracture displacement in doubtful situations, but the harmful effects of radiation should always be taken into account [103]. MRI can be useful for fractures involving the cartilaginous component, while ultrasound, in the hands of a skilled radiologist, can help with fractures of the lateral condyle in the youngest children [104,105].

### 4.3. Management

#### 4.3.1. Non-Surgical

Although various opinions have been proposed in the literature, up to which limit non-surgical treatment is tolerable, the opinion that stable fractures with a displacement of up to 2 mm and articular congruence can be treated non-surgically has become established [98]. Such fractures should be immobilized with the arm in a neutral position for six weeks, with a note that during the first three weeks it is necessary to carry out radiographic controls in order to avoid displacement, which may be an indication for surgical treatment. After removing the cast, it is necessary to start physical therapy until full activity [100,106,107].

Indications for surgical treatment are unstable fractures, fractures with displacement greater than 2 mm and unsuccessful non-surgical treatment. Surgical treatment can be performed via CRPP or via open reduction and internal fixation (ORIF) [98,108,109].

#### 4.3.2. Closed Reduction and Percutaneous Pinning

The indication for CRPP is fractures with a displacement of 2 to 4 mm (type II according to the Weiss classification) [98]. During adjustment, the elbow must be bent with a slight varus traction. As for K-wires, two were usually used, while more recently three are recommended, given that fixation with two wires can still allow fragment displacement. One pin parallel to the joint line and two oblique bicortical pins perpendicular to the fracture line are placed at an angle of 60°. Then the hand must be immobilized with the elbow bent to 90° and the forearm in a neutral position. Pins are usually removed after 4–6 weeks [110,111].

#### 4.3.3. Open Reduction and Internal Fixation

Indications for ORIF are fractures with a displacement greater than 4 mm, fractures with an incongruent articular surface, and fractures that cannot be resolved with CRPP. Among the approaches, posterolateral Kocher’s or Kaplan’s approach should be distinguished [98,111]. After the dissection, it is necessary to evacuate the usually present hematoma and clean the fracture surfaces with saline solution. After anatomical reduction, the fragment must be fixed with K-wires, as with percutaneous fixation, or with a cannulated screw through the nonarticular lateral portion of the lateral condyle and direct it toward the medial metaphysis for bicortical fixation or advance the screw into dense bone just lateral to the olecranon/coronoid fossae [112,113]. The arm needs to be immobilized with the elbow in a position up to 90° with the forearm in a neutral position. Extraction of osteosynthetic material is usually planned after 6–8 weeks, when it is necessary to start with physical therapy [114].

### 4.4. Complications

Among the possible complications, it is necessary to single out malunion and nonunion. If the surgeon does not perform an anatomical reduction of the bone fragment, malunion may occur, which can ultimately lead to post-traumatic arthrosis and *cubitus valgus* or *cubitus varus* [115]. Nonunion is defined as pseudarthrosis or the absence of bony union for more than 3 months after the injury. Several treatment methods have been proposed for treating a nonunion including osteosynthesis with bone grafting and pins or cannulated screws, osteosynthesis in situ, and closed reduction percutaneous fixation with a cannulated lag screw [116,117,118]. If there is a disturbance in the blood supply to the trochlea, it is possible to develop a complication called fishtail deformity, which is characterized by limited range of motion, stiffness, pain, and cubitus valgus deformity, which usually occurs 4–8 years after the injury. Some of the proposed treatment solutions are removal of loose bodies, epiphysiodesis, osteotomy, or ulnar nerve transposition [119].

## 5. Medial Condyle Fractures

Fracture of the medial condyle is a rare fracture with a peak incidence between the ages of 8 and 12, but it can occur earlier, and it usually occurs due to axial compression with concomitant valgus stress (fall on an outstretched arm), direct impaction of olecranon (direct fall on olecranon when the arm is flexed) and avulsion of the medial condyle due to violent contraction of the common flexor muscles of the forearm [95,120]. The specificity of medial condyle fractures is that the fractures are intra-articular and that the ulnar nerve passes in the immediate vicinity, which makes it vulnerable to injury [121,122].

### 5.1. Classification

Today, the most accepted classification when we talk about fractures of the medial condyle was brought by Kilfoyle, who classified fractures into three types (Table 6) [123].

### 5.2. Clinical Presentation and Radiographic Evaluation

A fracture usually presents with pain at the fracture site and swelling. Diagnosis can be difficult in younger children because the fracture can be difficult to see radiographically, and is usually misdiagnosed as a medial epicondyle fracture [123]. Consequently, fractures of the medial epicondyle with displacement in younger children who do not have an ossified trochlea should be examined with elbow stress in valgus (if unstable, the literature suggests stress radiographs or examination under anesthesia) [124]. Standard diagnostics include radiographic images in AP and lateral projection, and oblique projection may also be important. As with fractures of the lateral condyle, it must be kept in mind that the actual displacement is usually greater than on radiographs. Also, in doubtful cases, especially in younger children, an arthrogram, CT, MRI and ultrasound can be included in the diagnosis [120,122,124].

### 5.3. Management

#### 5.3.1. Non-Surgical

Indications for non-surgical treatment of medial condyle fractures are type I fractures and non-displaced type II fractures according to the Kilfoyle classification [123]. Such fractures should be immobilized for 4–6 weeks with the arm in a neutral position and with careful radiographic monitoring, including oblique projections, for the first three weeks. The literature also mentions a displacement limit of 2 mm as the limit after which surgical treatment should be started. After removing the cast, it is necessary to start with physical therapy [124].

#### 5.3.2. Surgical

The indication for surgical treatment is dislocated type II fractures and type III fractures according to the Kilfoyle classification, i.e., fractures with a displacement greater than 2 mm [123]. Surgical treatment includes open reduction with pinning or open reduction with screw fixation [125]. Which option the surgeon will choose depends on the size of the metaphyseal fragment. The fragment should be large enough for the screw [126]. Although both posterior and medial approaches are described, medial incision and anteromedial capsular dissection allow better exposure of the anterior articular surface and assessment of the quality of reduction [127].

### 5.4. Complications

Special attention should be paid to the complications that increase proportionally with the later recognition of the fracture. First of all, avascular necrosis should be singled out, considering that the blood supply of the trochlea can be disturbed during a fracture or during a surgical procedure, which the surgeon must take special care of [128]. Considering the anatomical location of the ulnar nerve, it should be taken into account that there is a possibility of its injury. Up to a third of medial condyle fractures can end in nonunion. There are no clear views on the treatment of nonunion of the medial condyle. While some surgeons advocate a conservative approach, others debate whether to perform open reduction with fixation or osteotomy [120,127,129,130]. Despite adequate surgical reduction, early physis closure and avascular necrosis of the epiphysis can be the causes of Fishtail deformity [131].

## 6. Medial Epicondyle Fractures

Injuries of the medial epicondyle most often occur between the ages of 9 and 14. They are often associated with elbow dislocations and radial neck fractures [132,133]. Due to its close proximity, the ulnar nerve is susceptible to contusion, neurapraxia from stretch, and entrapment within the joint [134]. Among the mechanisms of injury, a direct blow to the medial part of the elbow, an avulsion mechanism due to valgus stress, and a connection with elbow dislocation should be highlighted [132,135].

### 6.1. Classification

Although several classifications have been proposed by various authors, the most commonly used is the Watson-Jones classification, which classifies fractures of the medial epicondyle into four types (Table 7) [136,137].

### 6.2. Clinical Presentation and Radiographic Evaluation

Patients usually present with reduced range of motion with the elbow in flexion, pain and tenderness of the medial part of the elbow with swelling and sometimes ecchymosis. Special attention should be paid to the sensory and motor skills of the area innervated by the ulnar nerve [137,138,139]. As far as radiological diagnostics are concerned, the AP and lateral projections must be made, but it is definitely recommended to make an additional internal oblique projection, considering that the displacement of the medial epicondyle is extremely difficult to precisely measure based on basic radiographic images [140,141]. If there is ambiguity on the radiographic images, CT is definitely an option, despite the additional radiation. MRI will accurately determine the relationship between bone fragments and soft tissues inside the elbow in top athletes [142,143,144].

### 6.3. Management

#### 6.3.1. Non-Surgical

Unfortunately, there are still no clear guidelines when deciding on non-surgical versus surgical treatment. Knowing that radiographic images mostly do not correlate with real displacement, it is generally accepted that displacements of up to 5 mm on the AP projection can be approached non-surgically, but in addition to the above information, one should definitely take into account the mechanism of injury, the degree of stability of the elbow, the patient’s functional goals and the size of the displacement measured by of CT [145,146]. The arm must be immobilized for 3–4 weeks with the elbow held to 90°, with some advocating immobilization of the forearm in pronation. After removing the cast, it is necessary to start physical therapy. In most patients, even if the radiograph shows incomplete or fibrous union, the range of motion will be full [145,147].

#### 6.3.2. Surgical

Undisputed indications for surgery of medial epicondyle fractures are joint incarceration of the fragment, ulnar nerve entrapment or neuropathy, and open fractures, which are exceedingly rare. In modern practice, surgical treatment is also recommended for children with a displacement of more than 2 mm if they play top-class sports with high demands on the elbow [148,149]. Although there are various surgical approaches to the treatment of medial epicondyle fractures, the opinion that open reduction with cannulated screw fixation is the method of choice for fixation has recently become established [148,150]. It is important to note that there is a possibility of difficult reduction of the fragment, during which the arm must be flexed at the elbow, and the forearm placed in supination with the hand wrist flexed, while wrapping Esmarch bandage from the hand wrist to the elbow (milking maneuver) can additionally help with reduction [151]. It is necessary to place a screw that corresponds to the fragment. If the fragments are very small or comminuted, K-wires can be placed. During the surgical procedure, care must be taken all the time that the ulnar nerve is not within the fracture site, compressed by the hardware or subluxating out of the ulnar groove [149,151,152].

### 6.4. Complications

Complications include the possibility of incarceration of the fragment, which should be attempted to be freed by the Roberts maneuver (imparting a valgus force to the elbow, full extension of the wrist and fingers, and supination of the forearm to tension the medial epicondyle away from the joint). If you are not dexterous or the maneuver is performed difficult, you should definitely do an open reduction immediately and release the fragment [153]. If late nonunion is noticed, the most optimal treatment option is a bone grafting with screw fixation [154]. During the surgical procedure, there is a possibility of iatrogenic injury to the ulnar nerve, but the symptomatology may also be the result of local irritation of the nerve due to fibrous adhesions, as well as nerve entrapment. In most patients, symptoms disappear within 4–6 months [155,156].

## 7. Lateral Epicondyle Fractures

Like medial condyle fractures, lateral epicondyle fractures are extremely rare fractures, and are usually seen in conjunction with other fractures in the elbow. The peak of incidence is usually around the age of six. They are created by the action of avulsion forces of the extensor muscles of the wrist and forearm [157,158].

### 7.1. Clinical Presentation and Radiographic Evaluation

A clinical examination can usually verify pain in the lateral part of the elbow along with swelling as well as a limited range of motion. From the radiological diagnostic, it is necessary to do radiographic images in AP and lateral projection, and if necessary, internal oblique projection, which are usually sufficient for establishing a diagnosis and making a decision on the method of treatment. CT is usually required if there are associated injuries [100,158,159].

### 7.2. Management

Although there is no consensus on the magnitude of displacement that requires surgical reduction and fixation of the fracture, the absolute indication is an incarcerated fractured fragment within the elbow joint. Throughout the literature, the authors usually opted for closed or open reduction and fixation with K-wires or screw if the fragment was somewhat larger. In most fractures of the lateral epicondyle, the degree of displacement of the fracture fragment is minimal, and the injury heals after short-term immobilization for 3 weeks. Even if pseudoarthrosis occurs, most patients are asymptomatic [160,161,162].

### 7.3. Complications

Various complications of lateral humeral epicondyle fracture have been reported such as a block to extension, medial elbow dislocation and detachment of the medial epicondyle, physeal fractures and delayed union. Malunion of this kind of fracture in children has rarely been reported [1,163,164].

## 8. T-Condylar Fractures

T-condylar fractures are intra-articular, distal fractures of the humerus characterized by a central intercondylar split and extension of the fracture line through the medial and lateral column. They appear rarely in children with a peak of incidence around the age of thirteen, with a challenge in treatment [165]. Mechanisms leading to these fractures include falling on an outstretched arm with the elbow in flexion or falling directly on the elbow [166].

### 8.1. Classification

The first classification system for T-condylar fractures was described by Riseborough and Radin, then Jarvis and D’Astous and Toniolo and Wilkins [166,167,168]. Today, the most common AO (*Arbeitsgemeinschaft für Osteosynthesefragen*) classification is in use, which divides fractures into three types (Table 8) [169].

### 8.2. Clinical Presentation and Radiographic Evaluation

T-condylar fractures of the distal humerus usually cause intense pain with diffuse swelling of the entire elbow. Neurovascular status should be carefully assessed. Initially, it is necessary to take standard radiographic images in AP and lateral projection, and, if necessary, oblique projection. Some authors still advocate the use of intraoperative arthrography, but definitely the best modality of radiological diagnosis in the case of a T-condylar fracture is a CT scan with 3D reconstruction or MRI in younger children in order to perform optimal preoperative planning [170,171].

### 8.3. Management

#### 8.3.1. Non-Surgical

Indications for non-surgical treatment are undisplaced or minimally displaced fractures up to 2 mm, but they are extremely rare in this type of fracture. It is necessary to immobilize the arm for 3–4 weeks, with regular radiological follow-ups to prevent further displacement. After removing the cast, it is necessary to start physical therapy [165].

#### 8.3.2. Surgical

For displaced fractures, and since these fractures are intra-articular, closed reduction and immobilization will generally give worse results, so open reduction and internal fixation is an option [165,172]. Regardless of which open surgical treatment option the surgeon decides on, his goal must be to reconstruct the articular surface, align the distal humerus in the supracondylar region, and minimize complications such as non-healing and avascular necrosis of the trochlea [173,174]. Surgeons usually use a combination of cannulated screws and K-wires in the surgical treatment approach. Wires or screws are placed across the condyles transversely and then obliquely to the diaphysis, thereby reducing the condyles to each other first and then the condyles to the shaft. Some surgeons also decide to use plates in adolescents. The most standard configuration includes a medial and posterior lateral plate (90–90 dual plate construct). Among the approaches, the transolecranon approach and the Bryan-Morrey posterior triceps sparing approach should be highlighted [165,175,176,177]. After surgical treatment, it is usually necessary to additionally immobilize the arm in a cast for a minimum of 4 weeks before the patient begins physical therapy [173,174].

### 8.4. Complications

Given that these fractures are intra-articular, we always expect longer-term stiffness, and more serious complications include nonunion, osteonecrosis of the trochlea, ulnar neuropathy and heterotopic ossification [173,178,179].

## 9. Coronal Shear Fractures

Coronal shear fractures are rare injuries with a peak incidence around the age of twelve caused by the transmission of axial force through the radius head onto the capitellum and lateral ridge of trochlea, which can usually be caused by a fall on the outstretched hand with the elbow in extension or slight flexion [180].

### 9.1. Classification

Coronal shear fractures are classified into four types, each of which is associated with an eponym (Table 9) [180,181].

### 9.2. Clinical Presentation and Radiographic Evaluation

Coronal shear fractures present with significant pain and swelling around the elbow, and movements in the elbow are limited, especially flexion, given that the fracture fragment acts as a mechanical block. Although a plain radiograph is the initial radiological diagnostic, it can very often be misinterpreted, which can lead to a missed diagnosis. Usually, a fracture will not be seen on the AP projection, while a semilunar fragment displaced forward and superior (double arc or double bubble sign) can be seen on the lateral projection. If there is any additional doubt, it is necessary to do CT with 3D reconstruction or MRI in younger children [182,183,184].

### 9.3. Management

#### 9.3.1. Non-Surgical

Non-operative treatment is indicated for non-displaced fractures. The arm must be immobilized for a minimum of three weeks with regular weekly radiographic controls to prevent secondary displacement. After removing the cast, physical therapy is required [184].

#### 9.3.2. Surgical

With the development of headless screws, coronal shear fractures are surgically treated with open reduction and internal fixation. Optimal treatment is based on the anatomical reduction of the fragments, because even a minor displacement disturbs the congruity of the joint. Among the approaches that are applied, there are anterior, lateral and transolecranon, and through the literature the anterior has been imposed as the most optimal because it allows direct exposure of the fragment, followed by reduction of the fracture and anteroposterior fixation of the fracture with headless screws [185,186,187]. It is important to note that bioabsorbable headless screws should be implanted as much as possible in children. After stable fixation of the fracture, early mobilization in the framework of physical therapy is imperative [188,189].

### 9.4. Complications

Complications include arthritis at follow-up related to chondral injury, stiffness, avascular necrosis with possible consequent fishtail deformity and joint incongruity. Reconstructive options are limited to interposition or joint arthroplasty [180,190].

## 10. Proximal Radius Fractures

Fractures of the radial head and neck are relatively common fractures with peak incidence between the ages of nine and twelve [191]. The most common mechanism of injury is a fall on an outstretched arm and during anterior and posterior dislocations of the elbow [192].

### 10.1. Classification

For fractures of the proximal radius, there are the most classifications described so far, such as the Salter-Harris classification, Wilkins/Chambers classification, Judet classification, O’Brien classification, Mason classification, Bado classification, etc. [193,194,195,196]. In everyday clinical practice, the Judet classification is the most established (Table 10) [194].

### 10.2. Clinical Presentation and Radiographic Evaluation

If elbow luxation has not occurred, usually no deformity is noticed, but pain is present when attempting passive and active elbow movements, especially pronation and supination [197]. Plain radiographic images in AP and lateral projection are the first step in radiological diagnostic, if necessary supplemented by an oblique projection. On plain radiographic images, the most important thing is to evaluate the radiocapitellar line (RCL) and determine possible displacement and angulation in relation to it [24]. Although plain radiographic images are usually sufficient for diagnosis, additional radiological diagnostic allows ultrasound, arthrogram, MRI and CT for the purpose of additional preoperative planning, especially when associated elbow joint injuries are present [198,199].

### 10.3. Management

#### 10.3.1. Non-Surgical

Indications for non-operative treatment are Judet type I and type II fractures [194]. All larger angulations and displacements should be try reduced by closed reduction. As for the techniques, it is usually tried with pressure on the shaft of the proximal radius with the elbow in flexion up to 90° with the forearm in supination or pressure on the lateral epiphysis with the elbow at 90° during which the forearm rotates from full supination to full pronation (Israeli technique). Also described is the method with the help of an Esmarch bandage, which is tightly wrapped from the wrist to the elbow, which usually results in reduction of the fracture [200,201]. If you have an assistant, you can try to perform the Patterson maneuver (hold the elbow in extension and apply distal traction with the forearm supinated and pull the forearm into varus while applying direct pressure over the radial head) or the Nehar and Torch technique (elbow held in extension and supination with distal traction and varus force with assistant pushing laterally on radial shaft and surgeon pushing medially on radial head) [202,203]. If the closed reduction was successful, the arm must be immobilized for 3 weeks in a neutral position with slight pronation with regular radiographic monitoring once a week to prevent secondary displacement. After removing the cast, it is necessary to start with physical therapy [196].

#### 10.3.2. Surgical

If it is impossible to reduce the fracture to acceptable settings by closed reduction (angulation up to 30° and displacement up to 50% shaft diameter) surgical treatment with percutaneous or open technique is initiated [204]. As for percutaneous methods, fragment reduction is usually attempted with a K-wire. After the skin is pierced with the sharp end, the wire is turned, and the blunt end, with the help of fluoroscopy, tries to position the fragment by pushing [205]. The Metaizeau technique is also described for fragments that have bony contact and the angulation is not greater than 60°. This technique uses a K-wire or flexible titanium nail inserted retrograde from the distal radius. Under fluoroscopy, an attempt is made to capture the fragment with the tip of a wire or nail. After the fragment has been captured, the wire or nail must be rotated 180° to reduce the displacement and angulation [206]. If a satisfactory reduction cannot be obtained by any of the previous methods, open reduction is used. The most common reason is the existence of an interpositum. Using the Kocher, Kaplan, or Boyd approach, the fragment is accessed, which is anatomically reduced and fixed with two K-wires. After surgical treatment, the arm must be immobilized in cast for a minimum of 3 weeks with the elbow to 90° and the forearm in a neutral position. After removing the cast, it is necessary to start physical therapy [199].

### 10.4. Complications

Complications are the most common with the ORIF method, and are reflected primarily in the limited range of motion, especially rotation of the forearm. The possibility of malunion, radial head overgrowth, avascular necrosis, posterior interosseous nerve injury, fibrous adhesions, or radioulnar synostosis should also be mentioned [199,207,208,209].

## 11. Olecranon Fractures

Olecranon fractures in children are relatively rare with the highest incidence between five and ten years of age, and are usually associated with other injuries like radial head and neck fractures, distal radius/ulna fractures, fractures of the medial and lateral humeral condyles and Monteggia lesions. The most common mechanism is a fall on an outstretched arm or a flexed elbow [210].

### 11.1. Classification

Oleocranon fractures are divided into the more common short oblique and transverse fractures and the less common physeal and apophyseal avulsion fractures. Short oblique and transverse fractures are usually caused by falling on an outstretched arm with an extended elbow or by falling directly onto a flexed elbow. Physeal and apophyseal avulsion fractures most often occur in adolescents during sports activities [210,211]. Of the several classification systems, the most commonly used is Matthew’s, who divided oleocranon fractures into four types (Table 11) [212].

### 11.2. Clinical Presentation and Radiographic Evaluation

Children with oleocranon fractures usually present with swelling of posterior elbow and pain that is most pronounced when trying to extend the elbow. In the case of oleocranon fracture, plain radiographic images in AP and lateral projection are usually sufficient for diagnosis. The posterior fat pad sign in the lateral projection or longitudinal crack may indicate an occult fracture. If the oleocranon fracture is associated with other fractures, it is sometimes necessary to do CT or MRI for a detailed preoperative evaluation [210,213].

### 11.3. Management

#### 11.3.1. Non-Surgical

Indications for non-surgical treatment of oleocranon fractures are type I, II and III according to Matthew’s classification and displaced fractures up to 4 mm. It is necessary to immobilize the arm with an elbow at an angle of 90° and with the forearm in a neutral position for 3 weeks. In the case of minimally displaced fractures, it is necessary to perform radiological controls once a week to prevent secondary displacement. After removing the cast, physical therapy is required [214,215].

#### 11.3.2. Surgical

Tension band technique is the gold standard for transverse and oblique metaphyseal fractures. Upon access to the fracture, it is adjusted and held with reduction clamps. A transverse drill hole is made in the distal fragment (through which the steel wire will pass), and then the K-wires are placed from the posterior of the oleocranon through the fracture, which are bent in the proximal part so that the steel wire can be wound around them, which will ultimately form a loop in the shape of an eight. Instead of steel wire, the use of absorbable tension band suture is also an option [216,217]. In recent times, surgeons are increasingly using screw fixation instead of the tension band technique. After the reduction of the fracture, either by closed or open reduction, a guide wire for the cannulated screw is introduced through the triceps tendon in such a way that it engages the anterolateral cortex at least 2 cm distal to the fracture. A cannulated screw is placed bicortically to achieve compression [218]. For a comminuted fracture, it is necessary to apply the ORIF method, usually using a precontoured proximal ulna plate. For longitudinal fractures, the best option is open reduction and interfragmentary screws fixation [219].

### 11.4. Complications

Of the complications, hardware irritation should be singled out as the most frequent, then decreased range of motion, especially loss of full extension, and less often delayed union, nonunion, and ulnar nerve neurapraxia [220,221].

## 12. Elbow Dislocations

Elbow dislocations in children are not common, with a peak incidence between the ages of ten and fifteen, and usually occur in association with fractures or avulsion injuries, most often an injury to the medial epicondyle. The mechanism that most often leads to elbow dislocation is a fall on an outstretched arm [222].

### 12.1. Classification

Based on the presence or absence of associated fractures, dislocations are classified as simple or complex, and in relation to the direction of movement of the proximal radius and ulna in relation to the distal humerus, they are classified as posterior, anterior, medial, and lateral [223,224]. If the interosseous membrane between the proximal radius and the ulna is disrupted during the injury, then it is a divergent dislocation [225]. Due to the injury mechanism and anatomical predisposition, the most common form of dislocation is posterior [226].

### 12.2. Clinical Presentation and Radiographic Evaluation

In children with elbow dislocation, the deformity along with elbow swelling is obvious. Special attention should be focused on possible neurovascular injury. Plain radiography in AP and lateral projection is usually sufficient for diagnosis. When reviewing radiographs, special attention should be paid to possible associated injuries (medial epicondyle fractures, lateral condyle fractures, radial head and neck fractures, proximal ulna fractures, especially coronoid process fractures). If there is any doubt on plain radiographs, CT or MRI can be done, especially if osteochondral injury or soft tissue entrapment is suspected [222,223,224].

### 12.3. Management

#### 12.3.1. Non-Surgical

If there are no related fractures or other injuries, closed reduction is initiated, preferably as early as possible, because the progression of the swelling makes the whole technique more demanding [222,224]. Posterior dislocation is corrected with the forearm in a supinated position with the application of gradual longitudinal traction. To reduce the anterior dislocation, it is necessary to apply inline traction to the distal forearm with a posteriorly directed force on the forearm and an anteriorly directed force on the distal humerus. For medial and lateral dislocations, traction is applied first, followed by coronal plane deformity correction. After the reduction, it is necessary to assess the stability and possible blockage of movement caused by entrapment of soft tissues. Also, after a closed reduction, it is mandatory to repeat the radiographs in order not to miss an associated fracture, especially a fracture incarcerated in the joint. If there are no associated injuries, the arm should be immobilized for 1–2 weeks with the elbow flexed at 90° and the forearm in a neutral position. After removing the cast, it is necessary to start with physical therapy [227,228,229].

#### 12.3.2. Surgical

The indication for open reduction, apart from open dislocation, is usually unsuccessful closed reduction due to entrapment of bony fragments such as the medial epicondyle or osteochondral fragments or entrapment of neurovascular structures such as the medial nerve, ulnar nerve, or brachial artery [230,231]. The open approach primarily depends on the direction of the dislocation. If the capsule is preventing reduction, extension of the capsular rent allows reduction. Any constricting fascia more superficially should be released to assist with exposure and reduction. As for freeing the incarcerated fragment in the joint, the application of distraction and valgus stress is usually sufficient. All fractures in the elbow must be treated surgically according to the previously mentioned principles. Also, after surgical treatment, immobilization is initiated for 2–3 weeks, after which it is necessary to start with physical therapy [227,230,232].

### 12.4. Complications

The most common complication is elbow stiffness, especially in terminal extension. For the above reason, it is necessary to start physical therapy as early as possible. Heterotopic calcification, chronic instability with recurrent dislocations, and neurologic and vascular injuries are also possible [223,233,234].

## 13. Monteggia Fractures

Monteggia fractures are defined as proximal fractures of the ulna or plastic deformation of the ulna with associated dislocation of the radial head. The mechanism of occurrence is usually a fall on an outstretched arm with the forearm in pronation or direct trauma with the forearm in forced supination with a peak incidence between the ages of four and ten [235,236,237].

### 13.1. Classification

Monteggia fractures are classified according to the Bado classification in four types (Table 12) [238].

### 13.2. Clinical Presentation and Radiographic Evaluation

Monteggia fractures usually present with pain along with swelling and deformity, usually with the forearm in pronation. It is also important to check the neurovascular status. As far as diagnostic is concerned, a plain radiograph in AP and lateral projection, with the important note that the radiograph includes both the forearm and the elbow, is sufficient to establish a diagnosis that is often missed [239,240].

### 13.3. Management

#### 13.3.1. Non-Surgical

The focus of non-surgical treatment is on the reduction of the ulnar greenstick fracture or ulnar plastic deformation, which will also result in the reduction of the luxated radial head. After reduction, the arm must be immobilized with the forearm in supination with the elbow flexed to 100° for 6 weeks with regular radiographic controls to prevent secondary displacements. After removing the cast, it is necessary to start with physical therapy [241,242].

#### 13.3.2. Surgical

Monteggia fractures with a transverse or short oblique unstable ulna fracture are best treated with closed reduction and intramedullary fixation (with flexible titanium or stainless-steel nails or Kirschner wire in younger children), while patients with a long oblique or comminuted unstable ulna fracture are best treated with open reduction and internal fixation with plate and screw constructs (six- to seven-hole plate). The arm must be immobilized in a cast for 6 weeks, and the osteosynthesis material is usually removed after 3–6 months [242,243].

### 13.4. Complications

Complications after surgery for Monteggia fractures most often include non-union, malunion, nerve palsy, stiffness, and loss of range of motion. Symptomatic late-presenting cases can be treated successfully with reconstructive surgery (restoring ulnar length and alignment with possible open reduction of the radial head and annular ligament reconstruction) [244,245,246].

## 14. Conclusions

By respecting modern knowledge in the diagnostic and therapeutic approach to pediatric elbow fractures and dislocations, clinicians’ doubts and complications should be minimized to the satisfaction of children and their parents. Many new articles are published on the mentioned topic every day, which in the future will bring some new, probably even better scientific knowledge, all for even more optimal diagnostics and treatment.

## Figures and Tables

**Table 1:** Modified Gartland classification.

Type	Radiographic Findings	Stability
I	nondisplaced or minimally displaced (<2 mm), AHL intersects center of capitellum	stable
IIa	posterior cortex intact, AHL anterior to capitellum	stable
IIb	posterior cortex intact, AHL anterior to capitellum, rotational deformity	rotational instability
III	no cortical contact, AHL anterior to capitellum	unstable in extension
IV	no cortical contact, AHL anterior or posterior to capitellum	unstable in flexion and extension

AHL—anterior humeral line.

**Table 2:** Milch’s classification.

Type	Radiographic Findings
I	fracture line is lateral to trochlear groove
II	fracture line extends medially into trochlear groove

**Table 3:** Jakob’s classification.

Type	Radiographic Findings
Incomplete	fracture does not extend to the articular surface
Complete	fracture extends to the articular surface, no rotated fragment
Complete with rotation	fracture extends to the articular surface, rotated fragment

**Table 4:** Finnbogason classification.

Type	Radiographic Findings
A	fracture does not extend to the articular surface
B	fracture extends to the articular surface and is wider at the dorsolateral aspect of the humerus
C	fracture extends to the articular surface and is equally displaced at the articular surface and dorsolateral aspect of the humerus

**Table 5:** Weiss classification.

Type	Radiographic Findings
I	<2 mm displacement, intact cartilaginous hinge
II	2 mm–4 mm displacement, intact articular cartilage
III	>4 mm displacement, articular surface disrupted

**Table 6:** Kilfoyle classification.

Type	Radiographic Findings
I	nondisplaced fracture line extends to the physis
II	complete fracture through the epiphysis, no rotational displacement
III	severe displacement with rotation of the fragment

**Table 7:** Watson-Jones classification.

Type	Radiographic Findings
I	minimally displaced fragment
II	displaced fragment
III	displaced fragment entrapped in the elbow joint
IV	displaced fragment with a dislocation of the elbow

**Table 8:** AO classification of T-condylar fractures.

Type	Radiographic Findings
C1	intercondylar fracture without comminution
C2	fracture as one of split condyles with supracondylar comminution
C3	fracture as one of split condyles with articular surface comminution

**Table 9:** Classification of coronal shear fractures.

Type	Radiographic Findings
I (Hahn-Steinthal)	involves a large osseous portion of the capitellum in the coronal plane with little or no extension into the lateral trochlea
II (Kocher-Lorenz)	involves a shell of the articular cartilage with a minimal subchondral layer of bone
III (Broberg-Morrey)	comminuted or compression fracture
IV (McKee modification)	involves more than the lateral half of the trochlea

**Table 10:** Judet classification.

Type	Radiographic Findings
I	undisplacement or minimal displacement
II	lateral displacement <50% shaft diameter, angulation <30°
III	lateral displacement <100% shaft diameter, angulation 30°–60°
IVa	complete displacement, angulation 60°–80°
IVb	complete displacement, angulation >80°

**Table 11:** Matthew’s classification.

Type	Radiographic Findings
I	non-displaced without an associated injury
II	non-displaced with an associated proximal radius or distal humerus
III	non-displaced with an associated soft tissue injury
IV	displaced fractures (>4 mm)

**Table 12:** Bado classification.

Type	Radiographic Findings
I (extension type)	anterior dislocation of the radial head and fracture of the ulna with anterior angulation
II (flexion type)	posterior or posterior-lateral dislocation of the radial head and fracture of the ulna with posterior angulation
III (lateral type)	lateral or anterior lateral dislocation of the radial head with fracture of the ulna
IV (combined type)	anterior dislocation of the radial head with fracture of the proximal radius and ulna

## Data Availability

Not applicable.
